# Comparison of visual functions among early and late onset myopia

**DOI:** 10.1038/s41598-025-06778-0

**Published:** 2025-07-01

**Authors:** Salai Dhavamathi Janarthanan, Shonraj Ballae Ganeshrao, Kathleen Watt, Manali Hazarika, Ramesh S. Ve, Vijaya Pai H, Aiswaryah Radhakrishnan

**Affiliations:** 1 Department of Optometry, Manipal College of Health Professions, Manipal Academy of Higher Education, Manipal, Karnataka India; 2 Department of Ophthalmology, Kasturba Medical College, Manipal Academy of Higher Education, Manipal, Karnataka India; 3 School of Optometry and Vision Science, University of New South Wales, Sydney, Australia; 4 NeuraSim PVT LTD, Bengaluru, India; 5 Department of Optometry, SRM Medical College Hospital and Research Centre, Chennai, India

**Keywords:** High- and low-contrast visual acuity, Contrast sensitivity function, Blur perception, Depth of focus, Refractive errors, Visual system

## Abstract

Early-onset myopia (EOM) progresses more rapidly and sets a higher risk of developing high myopia, whereas late-onset myopia (LOM) is typically associated with lower refractive error. However, differences in visual function between these groups remain underexplored. This cross-sectional study compared high-contrast visual acuity, low-contrast visual acuity, contrast sensitivity function (CSF), blur perception, and depth of focus in individuals with EOM and LOM. Twenty participants (10 EOM, 10 LOM) aged 18–35 years with myopia ranging from − 0.50 to − 6.00 D were included. Visual function assessments were conducted using MATLAB and PsychoPy software, with all measurements taken under full-distance spectacle correction. While high-contrast and low-contrast visual acuity showed no significant differences, CSF parameters showed significant differences. The area under the curve, peak spatial frequency, and cutoff spatial frequency were significantly lower in EOM than in LOM (*p* = 0.019, 0.032, and 0.005, respectively). Blur perception thresholds also varied, though statistical significance was not reached. These findings suggest that individuals with EOM may show compromised contrast sensitivity, possibly affecting their overall visual quality. Understanding these functional differences can help in developing targeted interventions for myopia management, ensuring personalized approaches to optimize visual performance across different onset groups.

## Introduction

Myopia is one of the major public health concerns because of its high prevalence across the world. It is estimated that 49.8% of the world’s population will become myopic by 2050^1^. In India, the incidence of myopia is 7.5% in the 5–15-year-old age group^2^. Myopia can be classified based on etiology, degree of myopia, clinical entity, and age of onset. Age-related myopia can be classified into early-onset myopia (EOM) and late-onset myopia (LOM). EOM is defined as having the first contact lens or spectacle prescription before 16 years of age^3,4^, whereas LOM is defined as having the first optical correction at 17 years of age or older^5,6^. Understanding how visual functions differ between these groups is crucial for developing targeted clinical interventions and optimizing vision care. Comparing the visual functions of EOM and LOM individuals provides insight into accommodative responses, depth of focus, and overall visual performance, which can influence myopia progression and management strategies. As myopia incidence continues to rise worldwide, examining these differences is more important than ever for improving diagnostic and corrective approaches.

School-age myopia, or EOM, is affected by both gene expression and environmental factors^7^. In contrast, LOM is associated primarily with prolonged near-work and is often restricted to low or moderate levels of myopia^8^. Therefore, the extent to which genetics contributes to myopia development may vary depending on the type of myopia.

The visual functions of individuals should be considered when their vision-related abilities are assessed. The ability of the visual system to identify a stimulus is termed visual function. Visual functions include color vision, visual acuity, contrast sensitivity, blur perception, depth perception, stereo acuity and motion perception^9^.

Visual acuity is the visual function most assessed in routine clinical examinations that consider letters with high contrast. However, the world around us varies in color, contrast, shape, and clarity. Therefore, assessing only visual acuity is not sufficient, as a person may experience visual discomfort despite having 6/6 vision with the best-prescribed glasses. Contrast sensitivity refers to the ability to detect or identify changes in luminance between areas that lack well-defined boundaries^10^. Diminished contrast sensitivity is more psychologically concerning than decreased visual acuity is, as it significantly affects an individual’s ability to function in everyday life, impacts safety and mobility, compromises the quality of vision despite correction, and can signal potential progression of myopia^11^. Blur perception is a critical feature of our ocular system; it aids in focus and accommodation, enhances depth perception, directs visual attention, promotes visual comfort, helps assess optical quality, supports visual learning and development, and provides feedback for corrective measures. Blur perception has two important factors: blur discrimination, which indicates the amount of defocus necessary to perceive an already blurred image, and blur detection, which is the amount of defocus required to first perceive or detect blur^12^.

Assessment of visual functions provides deeper insight into the quality of vision. Studies have shown that despite having normal 6/6 vision, myopic individuals exhibit decreased contrast sensitivity, which further decreases as the degree of refractive error increases^13^. Also, myopic individuals tend to have increased blur thresholds, indicating decreased sensitivity to blur^14^. However, it remains unclear whether these reductions in visual function are influenced by the onset or duration of refractive error. Therefore, this study aimed to evaluate visual function in individuals with early-onset myopia and late-onset myopia. By comparing these two groups, we sought to better understand how the timing of myopia onset affects various aspects of visual function. In this study, we focused on the following visual functions like high and low-contrast visual acuity, quick contrast sensitivity function (qCSF), blur perception, and depth-of-focus (DOF).

## Methodology

This cross-sectional study was approved by the Institutional Research and Ethics Committee of Kasturba Hospital – Kasturba Medical College, Manipal Academy of Higher Education, Manipal (IEC number: 215/2021), and adhered to the tenets of the Declaration of Helsinki. All the subjects were recruited after written informed consent was obtained to participate in this study. Participants were recruited from the outpatient department of Kasturba Hospital, Ophthalmology Clinic, and from the student and staff populations of the Manipal Academy of Higher Education through advertisements. Participants aged 18–35 years with a spherical equivalent refractive error between − 0.50 D and − 6.00 D and astigmatism ≤ 1.50 DC were enrolled in the study. Based on the age of myopia onset, they were categorized into early-onset (7–16 years) and late-onset (17–35 years) myopia groups. Individuals with ocular comorbidities other than myopia, anisometropia greater than 1.00 D, binocular vision anomalies, or any developmental or genetic disorders were excluded from the study.

All participants underwent a routine clinical eye examination, which included visual acuity testing, subjective and objective refraction, slit lamp examination and fundus evaluation. A total of four experiments were conducted to measure high- and low-contrast visual acuity, depth of focus, quick CSF, and blur perceptions. All visual function tests were conducted under a uniform background luminance of 90 cd/m^2^, which falls within the photopic range and is consistent with previous studies on contrast sensitivity and acuity^15,16^. This level of luminance was selected to provide a comfortable and glare-free testing environment, while ensuring adequate stimulus visibility and minimizing retinal adaptation variability. The experiments were self-paced and conducted over multiple visits. They were all performed with full-distance spectacle correction.

### High and low-contrast visual acuity

**Fig. 1:**
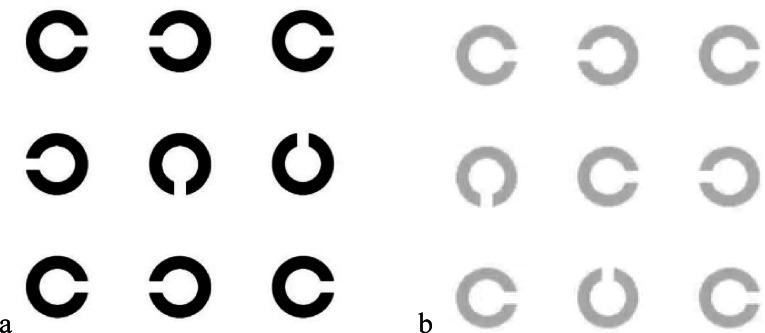
(**a**) High contrast visual acuity. (**b**) Low contrast visual acuity.

Visual acuity at high and low contrast levels was measured in logMAR using a custom-written MATLAB program. Participants were seated at a viewing distance of 3 m, which requires an accommodative effort of approximately 0.33 diopters. No additional plus lenses were used to compensate for this, as such a small demand is typically considered clinically insignificant. A four-alternative forced-choice (4AFC) task was employed, in which participants identified the orientation of a central Landolt C optotype surrounded by flankers (Fig. 1a,b). Letter flankers were used for crowding, as previous studies have shown that crowding effects are stronger with letter flankers compared to bar or box flankers^17^.

Visual acuity was measured using an adaptive staircase method. The experiment began with a 1.0 logMAR optotype size. For each correct response, the optotype size decreased by half a log unit, while for each incorrect response, the size increased by two log units. Final acuity thresholds were calculated by averaging the last four reversals.

### Contrast sensitivity function

The contrast sensitivity function (CSF) was assessed via a modified version of the quick CSF program^18^ in MATLAB. The qCSF utilizes a Bayesian algorithm to measure the CSF. The details of the algorithm have been explained in previous literature^18^. Briefly, participants completed a two-alternative forced-choice (2AFC) task in which they were asked to identify the orientation of a Gabor stimulus presented in one of two orientations. The spatial frequencies of the stimuli ranged from 1.0 to 50 cycles per degree (cpd). The contrast sensitivity function was measured using sinusoidal gratings presented in two oblique orientations: 45° (right-oblique) and 135° (left-oblique). Orientations were randomized across trials to minimize directional bias. The contrast and spatial frequency of the Gabor patch were dynamically adjusted based on the participants’ responses. For the obtained qCSF, the following parameters were estimated: the area under the curve, the cutoff spatial frequency, and the peak spatial frequency.

### Blur perception

To measure blur perception, participants were seated 1 m from a 24-inch, 60 Hz computer monitor (Dell). A headrest and chinrest were used to minimize head movement. Natural images, obtained from an open repository, were either blurred or sharpened by altering the slope of the 1/f spectrum.

**Fig. 2:**
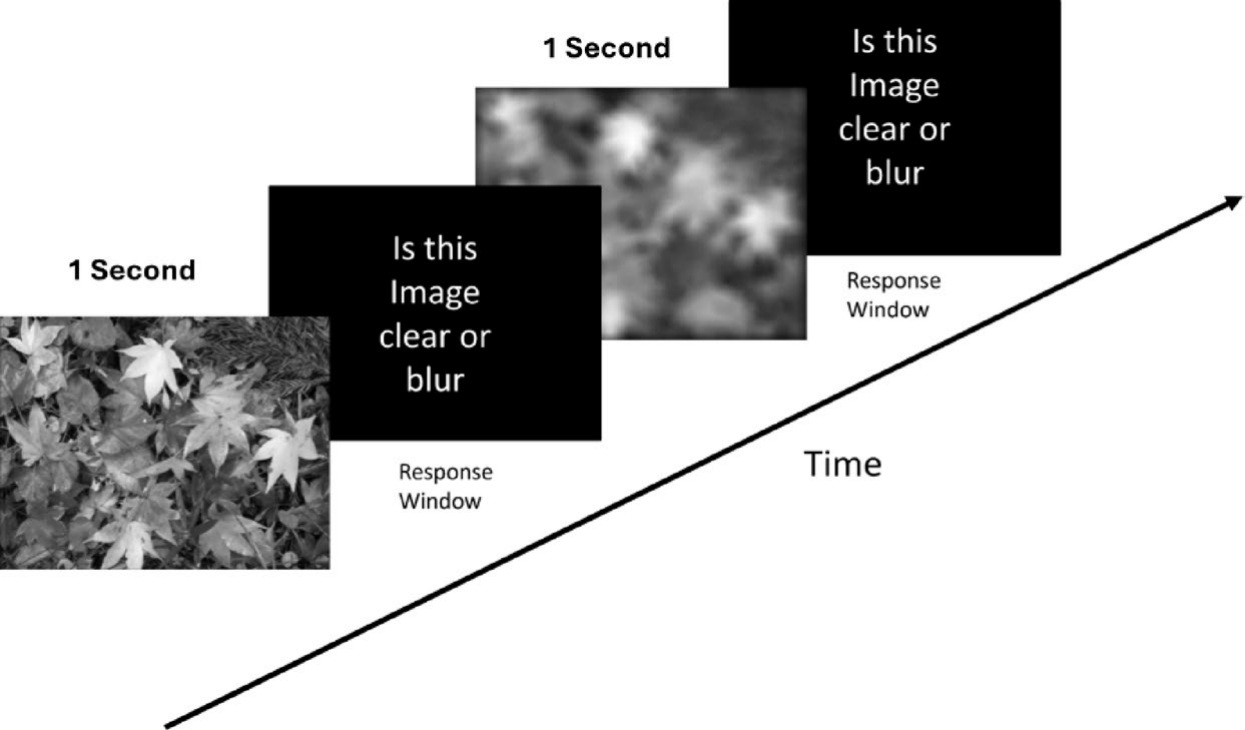
Blur perception experimental task.

Blur detection thresholds were measured using a two-alternative forced-choice (2AFC) task. In each trial, a single image subtending a 10-degree visual angle was presented on the computer screen, and participants were asked to indicate whether the image appeared clear or blurred. Each image was displayed for one second, followed by a response window; the next stimulus was presented only after a response was recorded (Fig. 2).

Two interleaved staircases were used: Staircase 1 started with a 0.1D blur level, and Staircase 2 with a 1D blur level. For each correct response, the blur level was reduced by half; for each incorrect response, the blur level was increased by half. Thresholds were calculated as the mean of the last four reversals of each staircase, and the final threshold was taken as the average of the two staircases.

### Depth of focus

Depth of focus (DoF) was defined as the range of defocus over which visual acuity did not deteriorate beyond 0.2 logMAR. This threshold was chosen based on previous literatures^19,20^ where a 0.2 logMAR criterion is commonly used to represent a functionally acceptable level of vision while accounting for inter-individual variability and the test–retest repeatability of visual acuity measurements. It reflects a clinically meaningful change in vision that remains within the functional range for daily tasks. Cycloplegia was induced using 1% cyclopentolate to eliminate accommodative fluctuations and ensure accurate measurement of true optical DoF. Controlling accommodation is particularly important in young adults and children, where active accommodation can significantly affect the defocus curve by artificially extending the perceived range of clear vision.

### Sample size

The sample size was calculated based on a previous study, where the standard deviation (SD) of depth of focus (DOF) was 0.13D^21^. With a Type I error set at 0.05 and a precision of 0.01, the estimated sample size was 10 participants per group.

### Data analysis

The data was analyzed using JASP software^22^. Normality was assessed with the Shapiro–Wilk test; if the p-value was greater than 0.05, the data were considered normally distributed. Independent samples t-tests or the non-parametric equivalent (Wilcoxon test) were used to compare the groups.

## Results

A total of 20 participants were enrolled (10 in the EOM group and 10 in the LOM group). The mean age was 21.1 (± 2.28) for the EOM group and 20.6 (± 1.35) for the LOM group. The average spherical equivalent refractive error was − 2.65 ± 1.39 DS in the EOM group and − 1.8 ± 1.2 DS in the LOM group. Except for the blur perception threshold, all other parameters were normally distributed. Independent t-tests revealed no statistically significant difference in age (*p* = 0.558) and no significant difference in refractive error (*p* = 0.168).

**Fig. 3:**
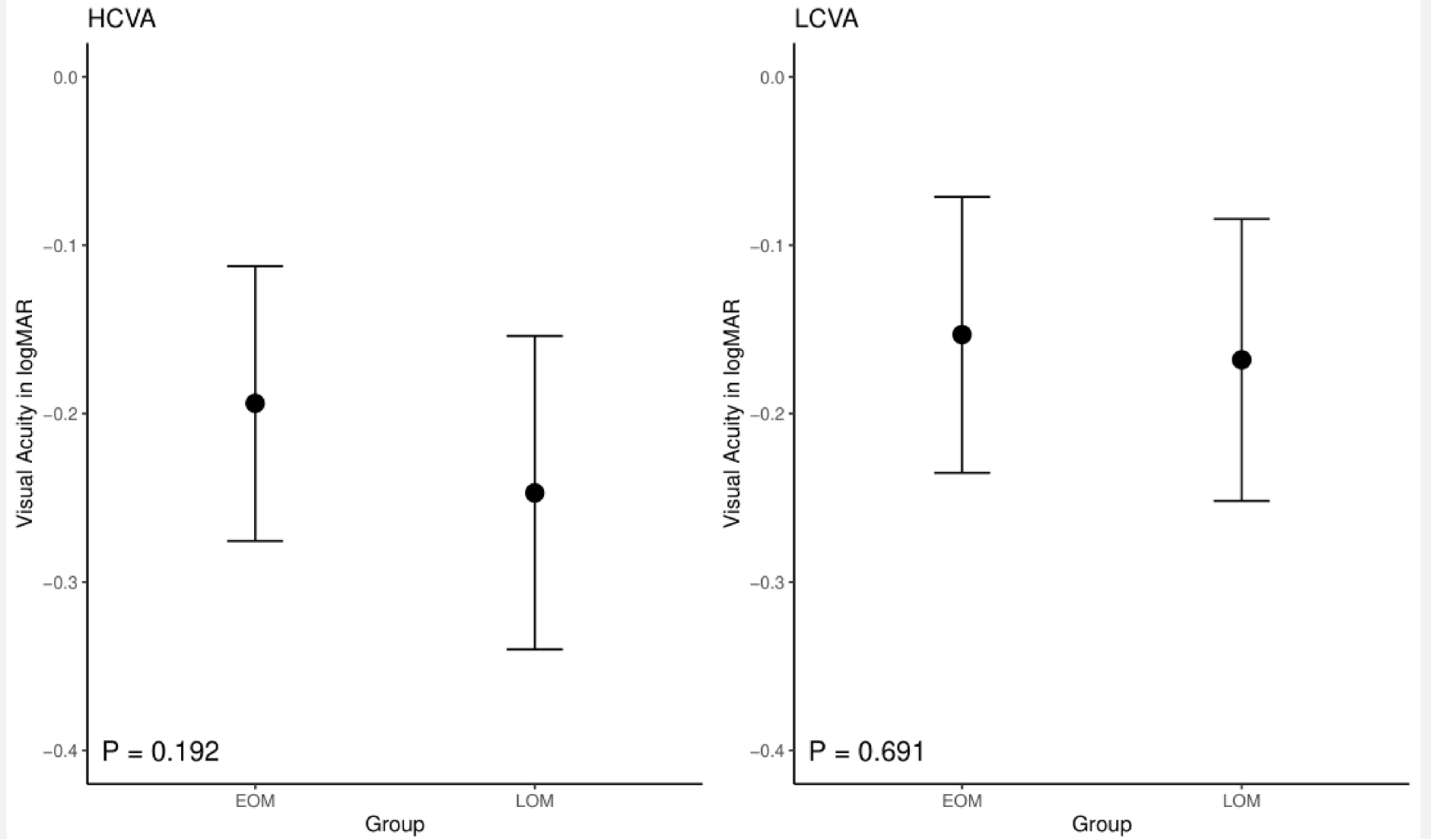
High contrast visual acuity (HCVA) and low contrast visual acuity (LCVA) in early-onset myopia (EOM) and late-onset myopia (LOM) patients. The dots represent the means, and the error bars represent the standard deviations.

The mean HCVA was − 0.19 ± 0.08 log MAR in the EOM group and − 0.247 ± 0.09 log MAR in the LOM group. The mean LCVA was -0.15 ± 0.08 log MAR in the EOM group and − 0.168 ± 0.08 log MAR in the LOM group. There was no statistically significant difference in HCVA (*p* = 0.192) or LCVA (*p* = 0.691) between the two groups (Fig. 3).

**Fig. 4:**
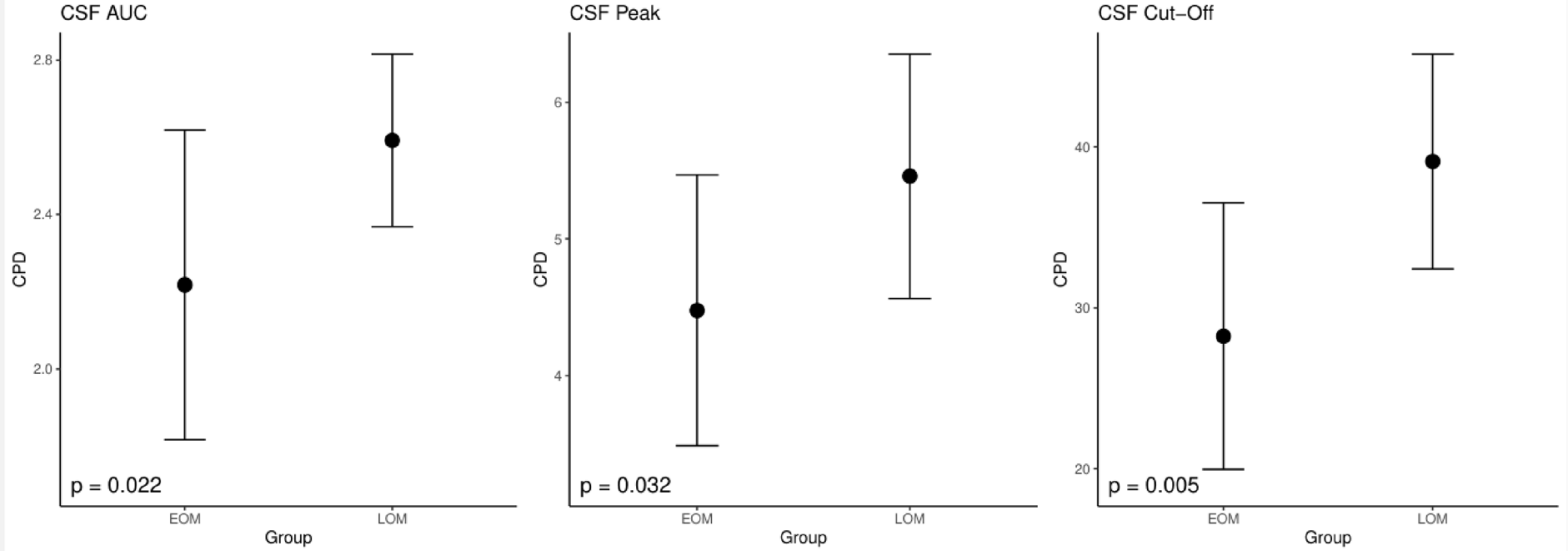
Area under the curve (AUC), peak contrast sensitivity function (CSF), and cutoff frequency of CSF in early-onset myopia (EOM) and late-onset myopia (LOM) patients. The dots represent the means, whereas the error bars indicate the standard deviations.

The analysis of CSF parameters revealed a significant difference between the two groups (EOM and LOM). The mean AUC value was 2.22(± 0.40) cpd in EOMs and 2.59 (± 0.22) cpd in LOMs (*p* = 0.022). Similarly, the peak CSF values were 4.48 cpd (± 0.99 cpd) in EOM and 5.46 cpd (± 0.89 cpd) in LOM (*p* = 0.032). The cutoff frequency was 28.24 cpd (± 8.28 cpd) in EOMs and 39.09 cpd (± 6.68 cpd) in LOMs (*p* = 0.005) (Fig. 4).

**Fig. 5:**
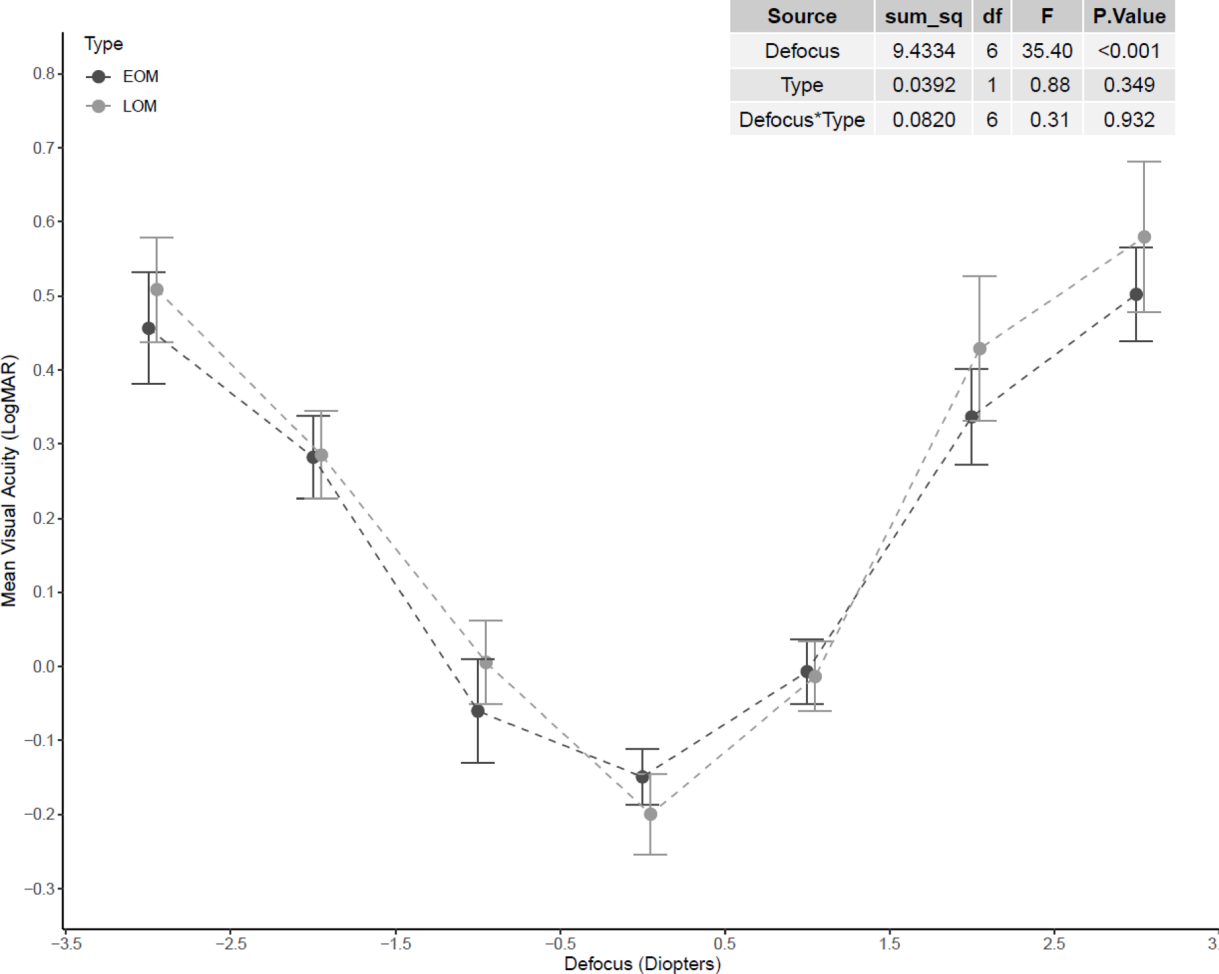
Visual acuity at different levels of blur for both early-onset myopia (EOM) and late-onset myopia (LOM). The dots represent the means, and the error bars represent the standard deviations.

The defocus curve analysis was conducted to evaluate the depth of focus (DoF) among individuals with early-onset myopia (EOM) and late-onset myopia (LOM). Mean visual acuity (logMAR) was plotted across a range of defocus levels from − 3.00 D to + 3.00 D. A two-way ANOVA revealed a statistically significant main effect of defocus on visual acuity (*F*(6, N) = 35.40, *p* < 0.001), indicating that visual acuity varied significantly across different levels of defocus. However, the main effect of myopia onset type (EOM vs. LOM) was not statistically significant (*F*(1, N) = 0.88, *p* = 0.349), suggesting no significant difference in visual acuity between the two groups. Additionally, the interaction between defocus level and myopia onset type was not significant (*F*(6, N) = 0.31, *p* = 0.932), indicating that the shape and slope of the defocus curves were similar across both groups (Fig. 5).

**Fig. 6:**
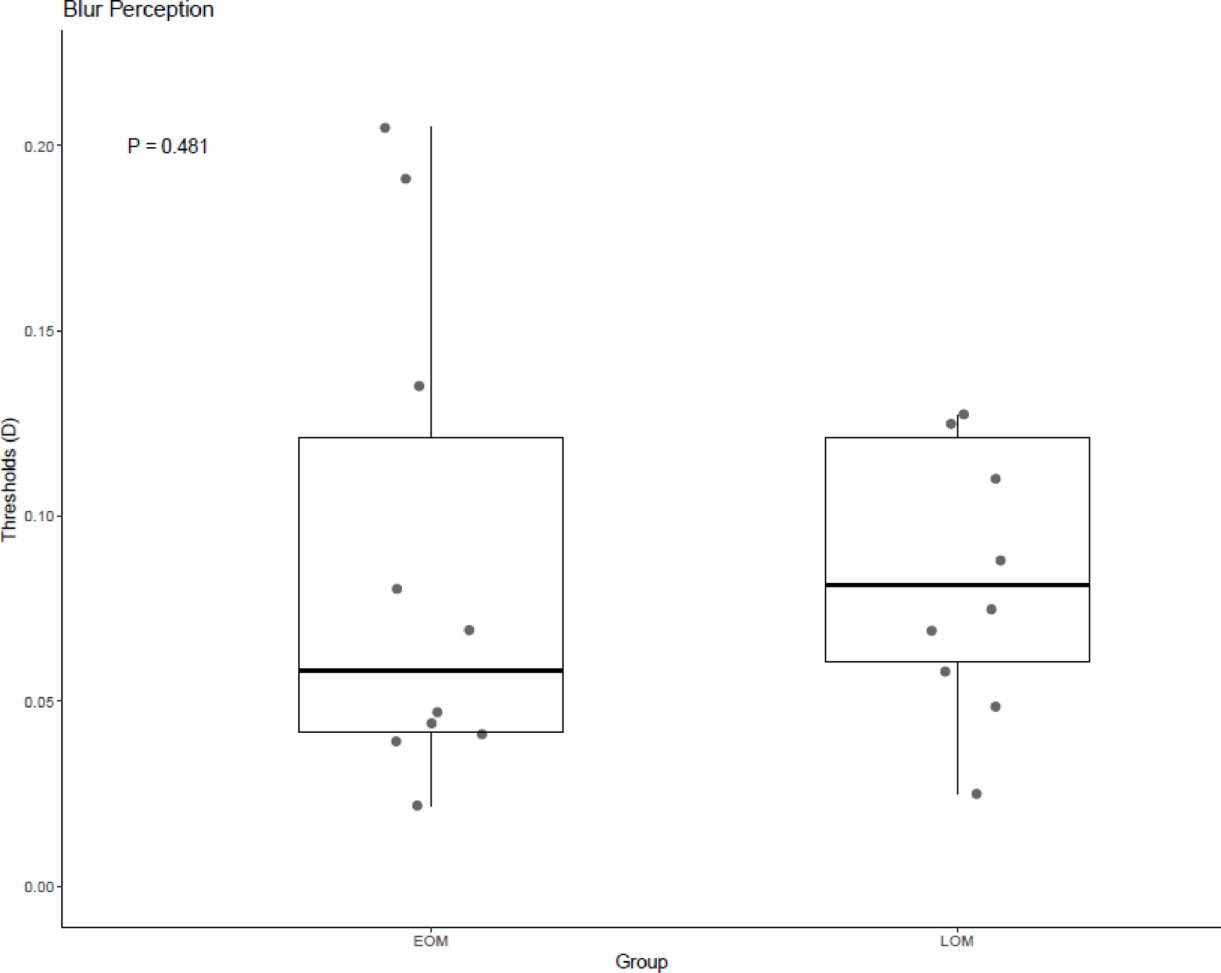
Boxplots showing the median and interquartile range of blur perception thresholds among early- and late-onset myopia patients.

The blur perception thresholds were non-normally distributed and are reported as median (IQR). The median threshold was 0.0583 DS (IQR 0.04–0.121) in the early-onset myopia group and 0.0815 DS (IQR 0.06–0.121) in the late-onset myopia group indicating no statistically significant difference (*p* = 0.481) (Fig. 6).

## Discussion

In this study, we investigated the visual functions of individuals with EOMs and LOMs. Our findings revealed no significant differences in high or low-contrast visual acuity, blur perception or depth of focus between the two groups. The only change observed was a change in the contrast sensitivity function.

Our results align with those of previous studies that have examined visual acuity in myopic individuals. Study by Huang et al.^14^ reported no significant differences in high- and low-contrast visual acuity between EOMs and LOMs. This consistency reinforces the view that the timing of myopia onset may not influence visual acuity under varying contrast conditions.

Lio et al.^23^ observed a reduction in contrast sensitivity function (CSF) associated with increasing myopic refractive error, attributing this decline primarily to higher optical aberrations and retinal image degradation in high myopia. Our study revealed a significant difference in CSF between early-onset and late-onset myopes despite no statistically significant difference in refractive error between the two groups. Individuals with early-onset myopia are exposed to blur or altered visual input at a more critical stage of visual development, which could lead to long-term changes in the cortical processing of contrast. Studies have suggested that the timing of myopia onset may influence the neural efficiency of the visual system, even if the optical characteristics remain similar. Also, differences in retinal or choroidal structure, which may not directly correlate with refractive error magnitude, could contribute to altered CSF. Early-onset myopes may have subtle anatomical or functional differences that impact contrast processing independently of spherical equivalent.

Studies have shown that myopia can affect contrast sensitivity in different regions of the retina. Li et al.^24^ assessed contrast sensitivity function in the parafoveal region and reported that myopia increased contrast sensitivity in superior and inferior visual field locations at 6° parafoveal and 12° perifoveal regions. These findings suggest that the impact of myopia on contrast sensitivity may vary depending on the retinal location. A recent study revealed that myopia is associated with a retinal deficit, particularly affecting ON pathways, leading to decreased contrast sensitivity^25^.

This study also aimed to compare the depth of focus (DoF) between individuals with early-onset myopia (EOM) and late-onset myopia (LOM) using defocus curve analysis. The results demonstrated a significant effect of defocus on visual acuity across all participants, consistent with previous literature reporting that optical defocus systematically degrades retinal image quality and visual acuity regardless of refractive status^19,26^. However, the absence of a statistically significant difference between the EOM and LOM groups suggests that the depth of focus is similar between the two groups.

Studies by Wang and Ciuffreda^27^ explored the effects of defocus on accommodative responses and visual acuity, reporting that variation in DoF is more closely related to individual accommodative behavior rather than the degree or onset of myopia. These findings support our observation that both the EOM and LOM groups exhibit comparable DoF characteristics.

Previous studies have shown that myopes generally exhibit a reduced depth of focus compared to emmetropes, potentially due to anatomical and optical factors such as larger pupil sizes and reduced retinal blur sensitivity^28,29^. While these studies observed variations in defocus tolerance between refractive groups, they did not specifically address the effect of onset age. Our findings extend this line of research by demonstrating that within the myopic population, early- and late-onset subgroups exhibit similar defocus profiles.

Furthermore, Zhou et al.^30^ emphasized the role of contrast sensitivity in influencing subjective depth of focus among progressive myopes, suggesting that visual function decline may be more closely related to myopia progression rather than onset age. Similarly, Liu et al.^31^ reported no significant differences in the slope of defocus curves among various subgroups of myopes wearing multifocal lenses, which align with our findings.

Our findings indicate that the mean blur perception threshold was slightly greater in individuals with late-onset myopia (LOM) than in those with early-onset myopia (EOM), though the difference was not statistically significant. This suggests that the ability to perceive blur does not significantly differ between these two myopic subgroups. One possible explanation is the presence of compensatory neural mechanisms or visual system adaptation to sustained myopic defocus over time, which may occur regardless of the age at myopia onset. The visual system’s ability to adapt to blur has been well-documented in the literature. Prolonged exposure to defocus can result in neural adaptations that affect contrast sensitivity and spatial frequency processing^32^. These neural adjustments may lead to an improved capacity to detect or tolerate blur, particularly in individuals exposed to myopic defocus over extended durations.

Individuals with EOM are typically exposed to myopic blur for a longer period due to the earlier onset of refractive error. This prolonged exposure may lead to enhanced neural adaptation and a heightened ability to detect subtle differences in optical clarity^33^. Supporting this, perceptual learning studies suggest that early and extended blur exposure enhances visual system sensitivity and discrimination ability^34^.

On the other hand, LOM individuals who develop myopia later may not have experienced sufficient duration of blur exposure to reach a similar level of neural adaptation. However, residual neural plasticity in this group could still enable some degree of blur adaptation, potentially explaining the absence of a significant difference between the groups^35^.

Furthermore, studies on blur adaptation have demonstrated that the visual system can recalibrate its response following sustained defocus. Rosenfield and Gilmartin^36^ reviewed that such adaptation can occur in both emmetropic and myopic individuals, suggesting a general plasticity in visual processing. A study by Cufflin et al.^37^ found that early-onset myopes showed greater increases in distal blur sensitivity thresholds following blur adaptation than late-onset myopes and emmetropes, indicating a heightened susceptibility or responsiveness to blur in EOM individuals.

Also, changes in ocular aberrations and accommodation responses with refractive error may influence blur sensitivity. Atchison et al.^38^ showed that higher-order aberrations vary with refractive error, which can affect retinal image quality and, consequently, blur perception. Gwiazda et al.^39^ also noted the role of accommodative behavior in influencing visual sensitivity in myopes, particularly in younger individuals. Moreover, the type of optical correction used may also influence an individual’s blur perception threshold. Janarthanan et al.^40^ reported that optical corrections used in myopia control, such as orthokeratology and multifocal lenses, can induce blur and lead to neural adaptation. Early-onset myopes, who often begin such treatments earlier, may develop greater sensitivity to defocus due to prolonged exposure, potentially influencing their blur perception. These findings suggest that blur perception is influenced by both the age of myopia onset and the visual system’s neural adaptation. The lack of a significant difference between groups indicates that both may have adapted sufficiently to offset the effects of onset timing.

### Implications for clinical practice

The differences in blur perception, CSF, and DoF between EOMs and LOMs suggest that management strategies should be tailored based on the onset of myopia. EOM individuals may require more precise optical interventions, such as orthokeratology or multifocal lenses, to address their lower tolerance to defocus and reduce CSF. LOM individuals may tolerate broader optical solutions and could benefit from strategies emphasizing visual comfort and long-term myopia stabilization.

### Limitations and future directions

Despite the insights provided, this study has several limitations that warrant consideration. The sample size was small, which may limit statistical power and the generalizability of the findings. The sample size in this study was primarily calculated for DoF and that this may not equally apply to other visual function parameters. While we observed significant differences in contrast sensitivity function (CSF), a larger cohort could help detect more precise differences in other visual functions and support subgroup analyses. In this study, contrast sensitivity was assessed using sinusoidal gratings at only two oblique orientations (45° and 135°). Although orientations were randomized, and conditions were consistent across groups, the use of oblique gratings may have influenced absolute sensitivity measurements due to the known oblique effect^41,42^. Future studies may benefit from incorporating a broader range of orientations, including cardinal and near-cardinal axes (e.g., 75°, 90°, and 105°), to better align with standard clinical or app-based CSF assessments.

We did not assess accommodation demand or amplitude of accommodation, which could have provided a more complete understanding of functional differences between early- and late-onset myopia (EOM and LOM). Since accommodation plays a key role in blur perception and depth of focus, its exclusion is a notable limitation particularly given that DoF was measured under cycloplegia, potentially masking intergroup variations in accommodative response.

Important optical and biometric factors such as axial length, corneal curvature, and higher-order aberrations were not measured. These variables can influence contrast sensitivity, image quality, and overall optical performance, and their absence limits our ability to fully interpret the underlying causes of the functional differences observed.

For future research, we recommend recruiting larger and age-matched cohorts to more accurately isolate the effects of myopia onset timing on visual function. Longitudinal studies should be conducted to track how visual performance evolves over time and to better understand developmental curves in early and late onset myopia. Incorporating objective measurements such as axial length, wavefront aberrations, and accommodative function would provide deeper insights into the optical and neural mechanisms underlying the observed differences. In addition, expanding the range of visual function assessments particularly through more detailed contrast sensitivity tests across various retinal eccentricities could help identify spatial and functional deficits with greater precision. Addressing these areas will allow future studies to build on our findings and contribute to a more complete understanding of how the timing of myopia onset influences both the quality and function of vision.

## Conclusion

In conclusion, this study found that individuals with early-onset myopia (EOM) exhibited significantly lower contrast sensitivity function (CSF) compared to those with late-onset myopia (LOM), despite having similar levels of refractive error. Other visual functions, including high- and low-contrast acuity, blur perception, and depth of focus, did not show statistically significant differences between groups.

These findings suggest that the timing of myopia onset may influence how the visual system processes contrast, potentially due to long-term neural adaptation or developmental factors. Clinically, this highlights the need to consider age of onset when designing optical interventions. EOM patients may benefit from more contrast-enhancing or targeted correction strategies, while LOM patients may have greater compliance to generalized solutions.

By differentiating visual function characteristics based on onset age, this study provides a step toward more personalized and functionally informed approaches to myopia management.

## Data Availability

Data availability can be stored in the repositories and can be provided upon request. The point of contact for data is Salai Dhavamathi J, email id is dhavamathi.j@manipal.edu.

## References

[1] Holden, B. A. et al. Global prevalence of myopia and high myopia and temporal trends from 2000 through 2050. *Ophthalmology* **123**(5), 1036–1042 (2016). 26875007 10.1016/j.ophtha.2016.01.006

[2] Agarwal, D. et al. Prevalence of myopia in Indian school children: Meta-analysis of last four decades. *PLoS ONE* **15**(10 October), e0240750 (2020). 33075102 10.1371/journal.pone.0240750 PMC7571694

[3] Mutti, D. O. & Zadnik, K. The utility of three predictors of juvenile-onset myopia: Child’s age, parent’s refractive error, and child’s refractive error. *Optom Vis. Sci.* **79**(7), 279–287 (2002).

[4] Goss, D. A. & Winkler, R. L. Progression of myopia in youth: Age of cessation. *Am. J. Optom. Physiol. Opt.* **60**(8), 651–658 (1983). 6624863 10.1097/00006324-198308000-00002

[5] Jiang, B. C. Parameters of accommodative and vergence systems and the development of late-onset myopia’. *Invest. Ophthalmol. Vis. Sci.* **36**, 8 (1995). 7601656

[6] Ravindran, M. et al. Prevalence and risk factors for myopia in a rural population in South India. *Acta Ophthalmol.* **92**(5), 483–488 (2014).

[7] Morgan, I. G. & Rose, K. A. Myopia: Is the nature-nurture debate finally over?. *Clin. Exp. Optom.* **102**(1), 3–17 (2019). 30380590 10.1111/cxo.12845

[8] Bullimore, M. A. et al. The Study of Progression of Adult Nearsightedness (SPAN): Design and baseline characteristics. *Optom. Vis. Sci.* **83**(8), 594–604 (2006). 16909085 10.1097/01.opx.0000230274.42843.28 PMC2760254

[9] Bennett, C. R., Bex, P. J., Bauer, C. M. & Merabet, L. B. The assessment of visual function and functional vision. *Semin. Pediatr. Neurol.* **31**, 30–40 (2019). 31548022 10.1016/j.spen.2019.05.006 PMC6761988

[10] Tidbury, L. P., Czanner, G. & Newsham, D. Fiat lux: The effect of illuminance on acuity testing. *Graefe’s Arch. Clin. Exp. Ophthalmol.* **254**(6), 1091–1097 (2016). 27106623 10.1007/s00417-016-3329-7 PMC4884565

[11] Sabel, B. A., Wang, J., Cárdenas-Morales, L., Faiq, M. & Heim, C. Mental stress as consequence and cause of vision loss: The dawn of psychosomatic ophthalmology for preventive and personalized medicine. *EPMA J.* **9**(2), 133–160 (2018). 29896314 10.1007/s13167-018-0136-8 PMC5972137

[12] Maiello, G., Walker, L., Bex, P. J. & Vera-Diaz, F. A. Blur perception throughout the visual field in myopia and emmetropia. *J. Vis.* **17**(5), 3 (2017). 28476060 10.1167/17.5.3 PMC5425112

[13] Stoimenova, B. D. The effect of myopia on contrast thresholds. *Investig. Opthalmol. Visual Sci.* **48**(5), 2371 (2007). 10.1167/iovs.05-1377 17460304

[14] Huang, Y. et al. Visual acuity, near phoria and accommodation in myopic children using spectacle lenses with aspherical lenslets: Results from a randomized clinical trial. *Eye Vis.* **9**, 33 (2022). 10.1186/s40662-022-00304-3 PMC9434851 36045391

[15] Owsley, C., Sekuler, R. & Siemsen, D. Contrast sensitivity throughout adulthood. *Vis. Res.* **23**(7), 689–699 (1983). 6613011 10.1016/0042-6989(83)90210-9

[16] Pelli, D. G., Robson, J. G. & Wilkins, A. J. The design of a new letter chart for measuring contrast sensitivity. *Clin. Vis. Sci.* **2**, 187–199 (1988).

[17] Pluháček, F., Musilová, L., Bedell, H. E. & Siderov, J. Number of flankers influences foveal crowding and contour interaction differently. *Vis. Res.* **179**, 9–18 (2021). 33271404 10.1016/j.visres.2020.11.002

[18] Lesmes, L. A., Lu, Z. L., Baek, J. & Albright, T. D. Bayesian adaptive estimation of the contrast sensitivity function: The quick CSF method. *J. Vis.* **10**(3), 17 (2010). 10.1167/10.3.17 PMC4439013 20377294

[19] Atchison, D. A. & Charman, W. N. Blur limits of the defocus curve in eyes corrected with spherical and multifocal lenses. *Optom Vis. Sci.* **83**(11), 777–783 (2006).

[20] Mirshahi, A., Chae, J. B. & Wu, Y. Through-focus visual performance with monovision and multifocal contact lenses: A systematic review. *Cont. Lens Anterior Eye* **44**(3), 101337 (2021).

[21] Labhishetty, V., Chakraborty, A. & Bobier, W. R. Is blur sensitivity altered in children with progressive myopia?. *Vision Res.* **154**, 142–153 (2019). 30472331 10.1016/j.visres.2018.11.002

[22] JASP Team. JASP (Version 0.19.3) [Computer software] (2024).

[23] Liou, S. W. & Chiu, C. J. Myopia and contrast sensitivity function. *Curr. Eye Res.* **22**(2), 81–84 (2001). 11402383 10.1076/ceyr.22.2.81.5530

[24] Xu, Z. et al. Assessing the contrast sensitivity function in myopic parafovea: A quick contrast sensitivity functions study. *Front Neurosci.* **16**, 971009 (2022). 36278008 10.3389/fnins.2022.971009 PMC9582454

[25] Poudel, S. et al. Contrast sensitivity of ON and OFF human retinal pathways in myopia. *J. Neurosci.* **44**(3), e1487232023 (2024). 38050109 10.1523/JNEUROSCI.1487-23.2023 PMC10860621

[26] Charman, W. N. & Jennings, J. A. The optical quality of the eye and its influence on visual resolution. *Vis. Res.* **16**(3), 289–296 (1976).1266073

[27] Wang, B. & Ciuffreda, K. J. Depth-of-focus of the human eye: Theory and clinical implications. *Vis. Res.* **46**(21), 3634–3642 (2006). 16414364 10.1016/j.survophthal.2005.11.003

[28] Atchison, D. A., Woods, R. L. & Bradley, A. Depth of focus of the human eye. *Clin. Exp. Optom.* **89**(3), 126–133 (2006).

[29] Radhakrishnan, H., Allen, P. M. & O’Leary, D. J. Depth-of-focus of the eye and its implications for presbyopia. *Ophthal. Physiol. Opt.* **33**(3), 314–323 (2013).

[30] Zhou, J., Zhang, Y., Jiang, B. & Wang, Q. Impact of contrast sensitivity on depth of focus in progressive myopes. *Sci. Rep.* **10**, 2251 (2020).32041963

[31] Liu, Y., Liu, Y., Zhu, S. & Zhang, H. Analysis of defocus curve in myopic subjects with and without multifocal contact lenses. *Optom. Vis. Sci.* **98**(7), 671–678 (2021).

[32] Wang, J., Zhuang, J. & Zhou, Y. Effects of prolonged blur exposure on contrast sensitivity and visual performance. *Ophthalmic Physiol. Opt.* **39**(7), 477–485 (2019).

[33] Chen, X., Wu, J. & Zhu, J. Neural adaptation to blur: A study on myopic and emmetropic individuals. *Sci. Rep.* **10**(1), 11245 (2020).32647181

[34] Saw, S. M., Matsumura, S. & Hoang, Q. V. Myopia development and perceptual learning: A review. *Clin. Exp. Optom.* **101**(5), 590–599 (2018).

[35] Logan, N. S. et al. IMI accommodation and binocular vision in myopia development and progression. *Invest. Ophthalmol. Vis. Sci.* **62**(5), 4 (2021). 33909034 10.1167/iovs.62.5.4 PMC8083074

[36] Rosenfield, M. & Gilmartin, B. Myopia and blur adaptation: A review of recent findings. *Vis. Res.* **157**, 34–42 (2019).

[37] Cufflin, M. P., Mankowska, A. & Mallen, E. A. Effect of blur adaptation on blur sensitivity and discrimination in emmetropes and myopes. *Invest. Ophthalmol. Vis. Sci.* **48**(6), 2932–2939 (2007). 17525230 10.1167/iovs.06-0836

[38] Atchison, D. A., Collins, M. J., Wildsoet, C. F. & Christensen, J. Measurement of monochromatic ocular aberrations of human eyes as a function of refractive error. *Optom. Vis. Sci.* **74**(8), 572–579 (1997). 10.1016/0042-6989(94)00139-d 7892727

[39] Gwiazda, J., Thorn, F., Held, R. & Bauer, J. Myopia and visual sensitivity: The role of accommodation. *Vis. Res.* **33**(13), 1717–1727 (1993).

[40] Janarthanan, S. D., Samiyullah, K., Madheswaran, G., Ballae Ganeshrao, S. & Watt, K. Exploring the impact of optical corrections on visual functions in myopia control-a scoping review. *Int. Ophthalmol.* **44**(1), 47 (2024). 38337138 10.1007/s10792-024-02937-w PMC10858094

[41] Appelle, S. Perception and discrimination as a function of stimulus orientation: The “oblique effect” in man and animals. *Psychol. Bull.* **78**(4), 266–278 (1972). 4562947 10.1037/h0033117

[42] Meier, K. & Giaschi, D. The development of contour integration: Evidence from psychophysics, electrophysiology, and functional imaging. *Front Psychol.* **5**, 824 (2014).25120522

